# 

**Published:** 1891-11-21

**Authors:** 


**BOVRIL**

In the "Lancet" of Nov. 11, 1865, **BARON LIEBIG says**:—"Were it possible to furnish the market at a reasona[illegible] price with a preparation of meat, combining in itself the albuminous together with the extractive principles, such a preparati[illegible] would have to be preferred to the Extractum Carnis, for it would contain all the nutritive constituents of meat." Again—"I ha[illegible] before stated that in preparing the Extract of Meat, the Albuminous principles rema[illegible] in the residue, they are lost to nutrition, and this is certainly a great disadvantage." **"BOVRIL"** contains the albumen and fibrine in the most perfect possible form, and those who know the requirements of the human system and the constituents of food, it will be apparent that this albumen a[illegible] fibrine is identical with what the body requires for recuperation, and that as a perfect form of concentrated nourishment it mu[illegible] supersede any animal aliment at present known.

SOLD THROUGHOUT THE UNITED KINGDOM.

### THE HOSPITAL

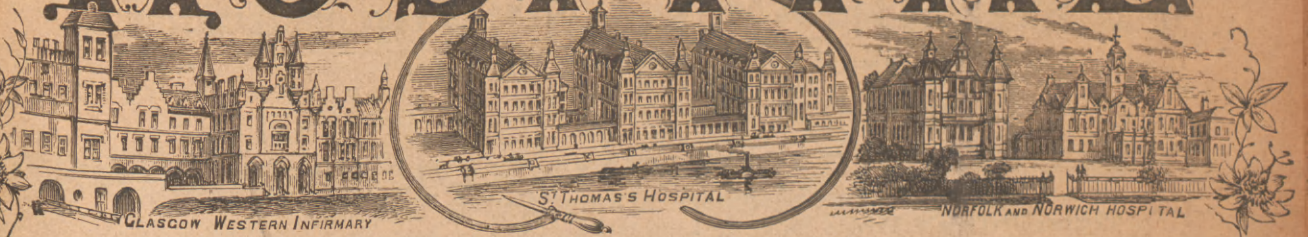

WITH EXTRA NURSING SUPPLEMENT.

No. 269. Vol. XI.] Saturday, November 21st, 1891. [One Penny.

### ST. DALMAS' "LEICESTER" SELVEDGE BANDAGES.

"Light, porous, elastic, and strong, and greatly superior to Calico."—*The Lancet.*

### "THE LEICESTER" POROUS PLASTERS on SCARLET FELT.

In one yard tins, 7½ in. wide, **Belladonna, Capsicum, or Opium Plaster,** 2/- per tin.
"We believe it to be one the best Belladonna Plasters ever made. It is very active, is not irritating, and gives plenty of support." —*The Lancet.*

### "THE LEICESTER" CAPSICUM PAPER.

More effectual than mustard plasters. In half yard tins, 5 in. wide, -/6 per tin; and one yard tins, in. wide, 1/- per tin.

### "THE LEICESTER" THORACIC and ABDOMINAL ROLLER BANDAGE (Selvedged),

7 in. wide, price 3/- per roll of 12 yards. A surgeon of large experience in accidents and midwifery, writes:—"Your new Selveged Wide Roller, of which I find 4 yards generally sufficient, is of great use in cases of Pleurodynia, Fracture of Ribs, and both before and after Parturition, in short, wherever broad firm support is required; midwives also appreciate it." "An efficient roller bandage has been brought out by Mr. St. Dalmas, of Leicester. It is seven inches broad, of strong porous material, not elastic, but permitting of slight stretching, light, and cheap. These qualities will recommend it to practitioners for the class of cases indicated, instead of the flannel roller in ordinary use."—*The Lancet.* "Mr. A. de St. Dalmas, of Leicester, has recently added to his list of useful rollers a new form of broad bandage. . . From a sample sent to us we have reason to believe that this broad bandage, the cost of which is moderate, will be found of service, both in private and hospital practice, as an efficient, and, at the same time, comfortable appliance."—*British Medical Journal.*

*The above can be obtained of all Wholesale and Retail Chemists.* *Special Quotations to Hospitals.*

---

### THE STUDENTS' OPHTHALMOSCOPE

**Is a Refraction Ophthalmoscope with 8 Lenses and Tilting Mirror and Large Bi-Convex Lens, in Neat Case, 15s.**

**Obstetric Vade-Mecum; Surgical and Electrical Instruments; Patented Batteries and Appliances, Belts, Stockings, &c.; Fisher's Patent Knee Caps and Bow Leg Splints; Bed Sheetings and Hospital and Nursing Requisites; Liquefied Gas and Inhaling Apparatus; Nitrous Oxide, Oxygen, and Hydrogen, &c.; Oxyhydrogen Lamp Depôt for Accumulators, for Cautery and Lighting, and for Re-charging, on the premises.**

**COXETER and SON, 4 and 6, Grafton Street, Gower Street, London, W.C.**

---

AN ADMITTED ANTIDOTE FOR INDIGESTION.

*Suits the most delicate constitution.*

*Sufferers from Indigestion may take it with impunity.*

### COOMBS' EUREKA AERATED PASTRY FLOUR.

Sold everywhere in 3d., 6d., 1s., and 2s. 6d. Bags.

SAMPLES SENT FREE to Matrons and Nurses in active work if Name of Hospital or Nursing Institute is sent with application to—

**COOMBS' EUREKA FLOUR CO., Ltd., 4, STANFORD STREET, NOTTINGHAM.**

